# RuO_2_ Nanorods as an Electrocatalyst for Proton Exchange Membrane Water Electrolysis

**DOI:** 10.3390/mi12111412

**Published:** 2021-11-17

**Authors:** Michael W. Cross, Richard P. Smith, Walter J. Varhue

**Affiliations:** 1 Electrical and Computer Engineering Department, David Crawford School of Engineering, Norwich University, Northfield, VT 05663, USA; mcross2@norwich.edu; 2 Mook Sea Farm, Nobleboro, ME 04555, USA; Richsmith1213@gmail.com; 3 Department of Electrical Engineering, College of Engineering and Mathematical Sciences, University of Vermont, Burlington, VT 05401, USA

**Keywords:** electrolyzer, electrocatalyst, nanorods, hydrogen production

## Abstract

A custom-built PEM electrolyzer cell was assembled using 6” stainless-steel ConFlat flanges which were fitted with a RuO_2_ nanorod-decorated, mixed metal oxide (MMO) ribbon mesh anode catalyst. The current density–voltage characteristics were measured for the RuO_2_ nanorod electrocatalyst while under constant water feed operation. The electrocatalytic behavior was investigated by making a series of physical modifications to the anode catalyst material. These experiments showed an improved activity due to the RuO_2_ nanorod electrocatalyst, resulting in a corresponding decrease in the electrochemical overpotential. These overpotentials were identified by collecting experimental data from various electrolyzer cell configurations, resulting in an improved understanding of the enhanced catalytic behavior. The micro-to-nano surface structure of the anode electrocatalyst layer is a critical factor determining the overall operation of the PEM electrolyzer. The improvement was determined to be due to the lowering of the potential barrier to electron escape in an electric field generated in the vicinity of a nanorod.

## 1. Introduction

The widespread use of renewable energy can be assisted by the development of a process to generate H_2_ by splitting water. The strict production of H_2_ from only renewable sources is referred to as green hydrogen [1]. The knowledge to produce hydrogen, in general, has been extensively pursued over the past 40+ years and continues as the need to replace carbon-based energy sources has become more urgent. The conversion of electrical energy into chemical energy through the generation of hydrogen for storage is an enabling technology for the widespread use of renewable energy. Inputs of harvested renewable energy and/or underutilized off-peak sources of electrical power can be better managed with the generation and storage of hydrogen. Electrolysis of water is the decomposition of water molecules into oxygen and hydrogen molecules powered by electricity. Efficient electrolysis has been achieved in electrochemical cells which increase performance through the use of a noble-metal electrocatalyst. Designing these electrocatalyst systems to be less material-dependent, more durable, and more efficient will support the conversion of electrical power into hydrogen as a fuel for energy storage. This study explores the use of RuO_2_ nanorod-decorated electrodes to enhance water electrolysis performed in a proton exchange membrane (PEM) electrolyzer cell. The minimum voltage theoretically required for the electrolysis reaction to occur is

(1) $$V_{min}=\frac{\Delta G_{d}}{nF},$$

where $\Delta G_{d}=237.2\;\frac{kJ}{mol}$ is the Gibbs free energy for the dissociation reaction at standard temperature and pressure (STP), the number of electrons transferred per molecule during water electrolysis, *n*, is equal to 2, and *F* is the Faraday constant. The resulting theoretical value of *V_min_* is 1.23 V. In practice, the voltage required is greater due to losses in the system and nonidealities in the electrochemical process. These additional voltage requirements are called overpotentials (*V_o_*) and can be determined experimentally by measuring the cell’s operational voltage (*V_op_*). This relationship is expressed in the following equation:

(2) $$V_{o}=V_{op}-V_{min}.$$

The expression for efficiency in terms of the minimum required voltage for an electrochemical cell is

(3) $$\eta_{v}=\frac{V_{min}}{V_{min}+V_{o}}.$$

Overpotentials occur for a variety of reasons including inactive electrocatalyst materials, increased electrical resistance between system components, and limitations imposed by the mass transfer of species. Lower overpotentials in a PEM water electrolysis system result in a greater overall efficiency of the cell.

In electrochemical reactions, the overpotentials are typically grouped into three main categories: activation, resistance, and mass transport. The greatest activation overpotentials in a water electrolyzer cell are experienced at the anode electrode, where the oxygen evolution reaction occurs (OER). Activation or reaction overpotential relates to chemical reactions that accompany electron transfer [2]. The activation energy or reaction overpotential can be significantly reduced with the use of an electrocatalyst such as RuO_2_ and IrO_2_ [3].

**Figure 1:**
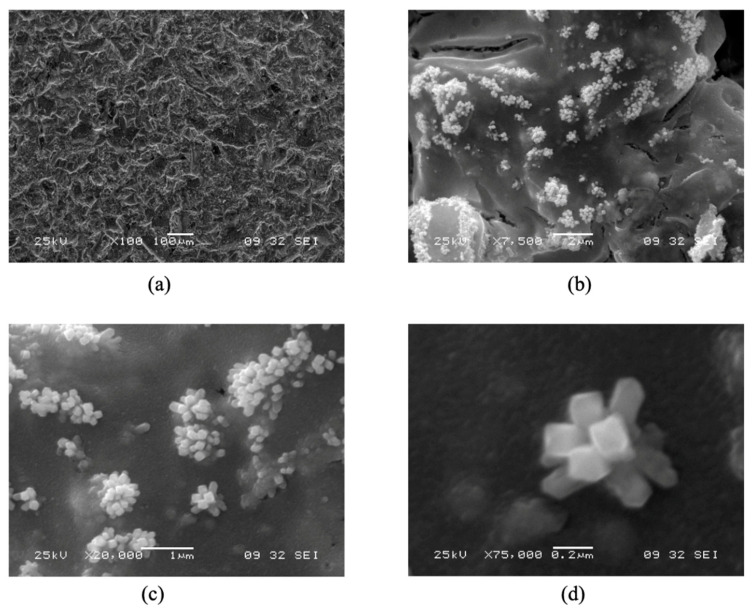
Varying magnification SEM images of RuO_2_ nanorods grown on a mixed metal oxide ribbon mesh anode. (**a**) RuO_2_ nanorods grown on MMO at 100× magnification, (**b**) RuO_2_ nanorods grown on MMO at 7500× magnification, (**c**) RuO_2_ nanorods grown on MMO at 20,000× magnification, and (**d**) RuO_2_ nanorods grown on MMO at 75,000× magnification.

The electrocatalytic material which is the focus of the present investigation is a RuO_2_ nanorod decorated ribbon mesh anode. The RuO_2_ nanorods are electrically conductive and transport electrons from the anode electrode to the H_2_O medium. The nanorods were grown on a solid conducting substrate surface, in this case, a mixed metal oxide (MMO) Telpro mesh material, by a reactive sputtering process. The nanorod-decorated surface effectively increases the interfacial surface area of the bare electrode, which is beneficial for the OER because it provides a large surface area [4]. Figure 1 shows scanning electron microscope (SEM) images of RuO_2_ nanorod-decorated surfaces like those used in these experiments. The nanorods can be grown longer than 1 μm and are controllably synthesized 10 to 1000 nm wide in the lateral dimension [5]. The increase in the interfacial area has been measured in previous experiments to be on the order of a factor of 10. This increase in surface area increases the number of possible reaction sites available on the catalyst surface [6].

A potentially more significant benefit of the RuO_2_ nanorod decoration, however, results from their sharp geometric features. This engineered material surface may have the ability to produce a high electric field locally at the electrode surface, which in theory may lower the required activation energy of the electrochemical reaction. The Stark effect states that an electric field has an influence on the quantum potential well, resulting in a change in symmetry of the free-energy barrier and an increased ability for electrons to escape the potential well [7,8]. When electrically biased, a high-field region forms locally around the exterior of the rectangular nanorods. The magnitude of this electric field is inversely proportional to the size of the nanostructure; therefore, a high field strength is expected for the nanorod. This enhanced field, induced by the geometry of the nanorods, is expected to decrease the reaction overpotential, resulting in increased electrocatalytic ability.

## 2. Methods and Materials

### 2.1. PEM Modifications

Through a combination of controlled experiments, modifying the PEM cell configuration, and using different electrocatalyst materials, a characterization of the anode electrocatalyst material was performed. The kinetics of the electrochemical reactions was monitored by measuring the electrical current provided to the anode electrode as a function of the applied voltage (*V_op_*). The relationship between the electrical current (*J_o_*) and applied voltage potential (*V_op_*) permits the overpotentials to be determined. This equation, the Butler–Volmer equation, is derived from basic principles of thermodynamic equilibrium and heterogeneous chemical kinetics. 

The starting, unmodified PEM cell consisted of a graphite bipolar plate functioning as the cathode, a purchased multiple electrode assembly (MEA), and a custom-made stainless-steel bipolar plate that was used as the anode. The graphite cathode bipolar plate had a serpentine channeled flow field (5.18 cm^2^ surface area, 0.1 cm deep). The stainless-steel anode had an open gas flow field (4.95 cm^2^ surface area, 0.2 cm deep), which provided space to insert additional electrocatalyst structures. The PEM electrolyzer device was operated as a water electrolyzer, and the collected *I* vs. *V* data were used to generate Tafel plots for further analysis. All MEAs used throughout the investigation had identical cathode assemblies, i.e., the cathode was loaded with 4 mg/cm^2^ of platinum black (PtB) and covered with a carbon cloth gas diffusion layer (GDL). The ion exchange membrane used for all MEAs was Nafion 115, which was 127 μm thick.

### 2.2. Fabricating RuO_2_ NRs

The inclusion of the new RuO_2_ nanorod electrocatalyst into this cell was complicated. Although it would have been preferable to compare two similar PEM electrolyzer cells that differed only by the composition of the anode electrocatalyst material contained in their MEAs, this was not possible. The catalyst material of interest in this investigation, RuO_2_ NRs, requires deposition on a solid substrate surface at a high temperature (500 °C). The nanorods were deposited under very specific process conditions of substrate temperature, gas composition, and power to the sputter gun. In these experiments, the nanorods were deposited for 30 min in 5% Ar/O_2_ plasma [4,5]. The commercially obtained MEAs fabricated on a Nafion substrate could not be used as the substrate because these would be destroyed at these high processing temperatures.

**Figure 2:**
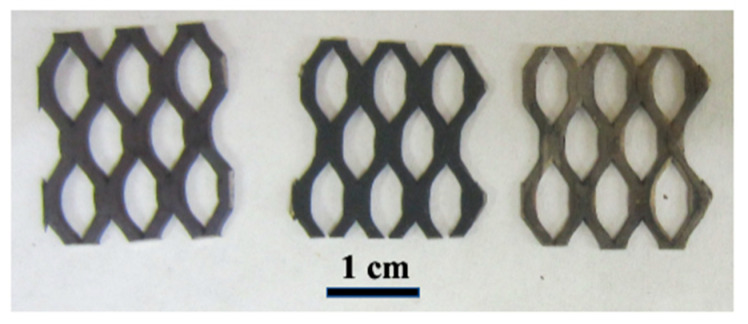
Various anode electrodes compared. From left to right: RuO_2_ nanorod-decorated MMO ribbon mesh anode, Ir/Ta MMO ribbon mesh anode, and Ti ribbon mesh anode.

To deposit the RuO_2_ NR electrocatalyst material into the MEA structure, a thin film of RuO_2_ NRs was deposited using a reactive sputtering process onto a purchased Telpro MMO ribbon mesh. The Telpro ribbon has a base of Ti and is electroplated with Ir/Ta catalyst coating. A photograph of Telpro MMO ribbon electrodes is shown in Figure 2 below.

**Figure 3:**
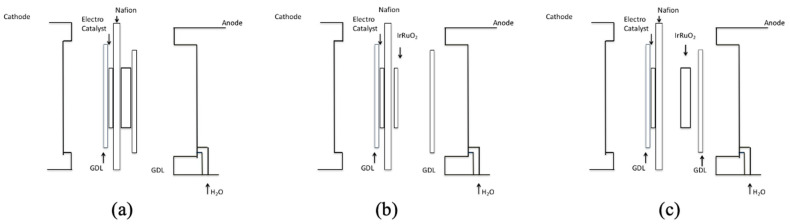
(**a**) PEM electrolyzer with as purchased intact MEA, (**b**) PEM electrolyzer with detached GDL, and (**c**) PEM electrolyzer with detached anode electrocatalyst (IrRuO_2_) and GDL.

Two samples of the Telpro MMO ribbon mesh anode were prepared and tested for comparison. Each piece was cut into a 2.2 cm by 2.1 cm sample, and the edges were sanded to eliminate possible damage to the MEA or GDL. One sample was left alone, as the MMO acts as an Ir/Ta electrocatalyst material layer. The second sample consisted of a Telpro MMO ribbon mesh anode decorated with a RuO_2_ NR layer. It was necessary to first measure the effects of disassembling the MEA before assessing the effect of the nanorod electrocatalyst material. This comparison was made by disassembling the anode side of the originally purchased MEA system and measuring the performance. The effect that each of these physical modifications made to the anode side on the PEM electrolyzer was measured and analyzed. A sketch showing the disassembly of the original PEM Electrolyzer MEAs is shown in Figure 3a–c. 

**Figure 4:**
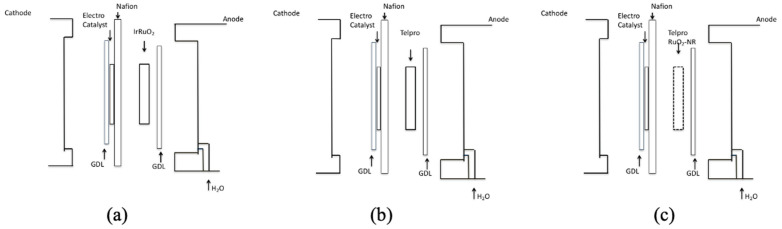
(**a**) PEM electrolyzer with detached anode electrocatalyst (IrRuO_2_) and GDL, (**b**) PEM electrolyzer with detached anode electrocatalyst (Ir/Ta on Telpro) and GDL, and (**c**) PEM electrolyzer with detached anode electrocatalyst (IrTa and RuO_2_ NRs on Telpro) and GDL.

Next, three different cell modifications were used to determine the effect of the RuO_2_ nanorod electrocatalyst when used to form the anode side of the PEM electrolyzer (see Figure 4).

To be clear, this was physically achieved by purchasing a MEA structure with nothing attached on the anode side or a bare Nafion membrane surface. 

The effect that each modification described above had on the operation of the PEM electrolyzer was determined by measuring the *I* vs. *V_op_* operation of the electrolyzer. To ensure proper hydration of the Nafion membrane, the electrolyzer cell was flushed with water for at least 1 h before voltage was applied. The US Fuel Cell Council (USFCC) protocol and additional operational protocols for the successful operation of a PEM electrolyzer were utilized. In our experimental procedure, we endeavored to meet or exceed these recommended protocols [9], although a period of 1 h was chosen for the sake of consistency. An additional voltage preconditioning period was followed by connecting the power supply to the electrolyzer cell, setting digital multimeters (DMMS) to measure the current passing through the cell and the voltage potential between the cells, and applying a voltage of 2.5 V. The USFCC protocol, which calls for a 1 h period of constant applied voltage at 1.8 V [9], was used as a benchmark to develop a 1 h at 2.5 V preconditioning process which provided consistent operation. These preconditioning periods provided time for the electrolyzer cell to achieve steady-state operation.

At the end of the 2 h preconditioning period of the electrolyzer cell, an initial reading was taken, and then the voltage was set to 2.5 V. The voltage was swept by a 0.2 V initial decrease from 2.5 V to 2.1 V and then a 0.1 V decrease from 2.1 V to 0.8 V. A final reading was taken at 0.6 V. Sixty seconds were allotted between each voltage increment to allow for the steady-state condition to exist. If the current was still flowing when 0.6 V was applied, an additional 0.2 V drop was made until the current read 0 A. Once complete, the electrolyzer cell was disassembled layer by layer, noting any physical changes that could have occurred during the operation of the cell.

The results produced a Tafel plot which was used to determine the activation overpotential occurring in the system. Parameter values were determined by graphically analyzing the resulting Tafel plot slope. Equations for the charge transfer coefficient and exchange current density were applied to characterize the observed electrochemical behavior. A single change in the cell configuration was then quantified by the change in the value of the corresponding overpotential parameters. The Tafel equation (4) was obtained from the Butler–Volmer equation. The derivation further assumes that the reaction at the cathode does not contribute significantly to the overpotential; therefore, the Tafel equation can be simplified to only consider the reaction at the anode [10].

(4) $$J=J_{o}exp\left[\frac{V_{o}\alpha F}{RT}\right].$$

The Tafel equation above includes two principal parameters: the exchange current density, *J_o_*, and the charge transfer coefficient, *α*. The exchange current density, *J_o_*, is the current flow resulting from the redox of the aqueous electrolyte species on the electrocatalyst surfaces. The reaction rate is dependent upon the rate constant and surface concentrations of the electrochemical reactants [7]. A large exchange current density represents a high reactivity of the electrode [11]. The charge transfer coefficient, α, is dependent on the electric field strength present at the electrode–reactant interface, which influences the quantum potential energy barrier for the reaction complex [10]. A change in *α* affects the slope of the exponential *I* vs. *V* curve. These two parameters are used to express the overpotential that is measured for different electrolyzer cell configurations, thus permitting a comparison of different electrocatalyst materials.

## 3. Results and Discussion

Through a combination of controlled experiments on the operation of a custom-built PEM electrolyzer cell, the effects of varying cell configuration and the use of different electrocatalyst materials were determined. The disassembly of the existing MEA structure resulted in additional resistances to current flow and impediments to ion diffusion. To ultimately understand and assess the performance of a new engineered electrocatalyst material, the effect of disassembling the original MEA structure must first be understood. A brief discussion of the effects of each modification is presented below.

The cell operation was plotted as ln (*J_o_*) vs. *V_o_*, known as the Tafel plot. These curves were used to evaluate the overpotential parameters, (*J_o_*) and (*α*), which were defined in Equation (4) above. The exchange current density (*J_o_*) is related to the number of surface sites available for the electrolysis reaction, while the charge transfer coefficient (*α*) represents the effect of the electric field strength on the shape of the activation complex barrier [8,10]. 

### 3.1. Separation of Gas Diffusion Layer from Electrocatalyst Surface

**Figure 5:**
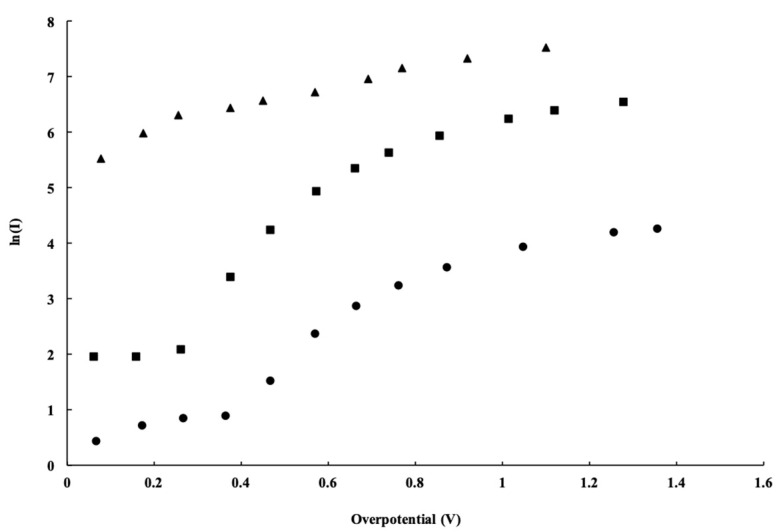
Tafel plot for the custom-built PEM with fully assembled MEA (solid triangle data), a fully detached GDL (solid circular dots), and a repositioned GDL with a Telpro ribbon mesh inserted between the electrocatalyst surface and the GDL (solid square dots).

In the first modification, the placement of GDL was considered, either attached directly to the electrocatalyst surface of the MEA or positioned as a separate layer. Analysis of the water electrolysis *J*–*V_o_* plot (Figure 5) indicates that the fully assembled MEA had the greatest overall performance. The overall effect of the separation of the GDL from the MEA suggests that the separated GDL experienced overpotential likely caused by increased electronic resistance between the electrocatalyst surface and the anode bipolar plate. The effect of this separation is important to consider because disassembly of the commercial five-layer MEA is required to test the electrocatalyst materials of interest.

The electronic separation of the GDL from the electrocatalyst surface shows that a large series resistance was incurred. The installation of the conducting Telpro ribbon mesh anode catalyst structure should have significantly eliminated this resistance. This is in fact what was observed in Figure 5 (solid square dots), which shows the measured current density of the cell once the conducting Telpro ribbon mesh was incorporated. This provided a path of lower resistance between the GDL and electrocatalyst surface. 

### 3.2. Separation of Electrocatalyst Surface from Nafion Membrane

The Nafion membrane is an electrical insulator but, in theory, an ionic conductor. The experimental results presented and discussed in this section result from separating the electrocatalyst material from the Nafion membrane surface. The ability to exchange nonattached electrocatalyst material layers is required to investigate the electrocatalytic behavior of the RuO_2_ nanorods. 

The effect of a separation between the electrocatalyst surface and the Nafion membrane surface will decrease the cell’s overall performance and introduce additional overvoltages to the Tafel plot. Hydronium ions produced in the electrocatalyst material must diffuse to the Nafion membrane, where they will drift under the applied field toward the cathode side of the cell. This becomes a limiting factor of the water electrolysis reaction for this cell. 

Previous work reported by Wilson and Gottesfeld [12] discussed the significance of good contact between the Nafion membrane and the electrocatalyst material. In fabricating the PEM cell, the anode electrocatalyst material should be coated on the Nafion membrane surface. This will minimize any separation between the Nafion membrane and the electrocatalyst material. The transport of hydronium ions by both diffusion in the gap between the layers and drift in the Nafion membrane results in significant overvoltage contributions of the electrolyzer cell relative to the transport of electrons [13]. 

### 3.3. Anode Electrocatalyst Materials

**Figure 6:**
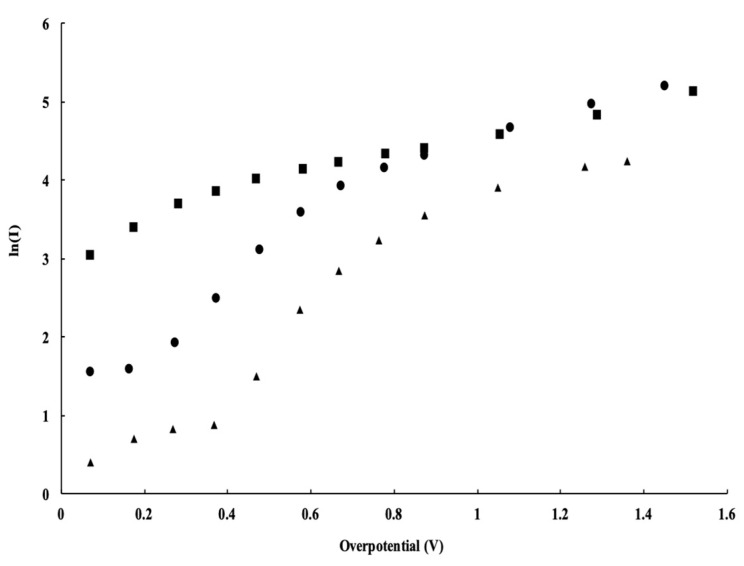
Tafel plot for the custom-built PEM used in this investigation with three different anode electrocatalyst materials including a detached electrocatalyst layer with IrRuO_2_ (solid triangle symbols), Telpro ribbon mesh coated with RuO_2_ nanorods (solid circular symbols), and Telpro ribbon mesh with Ir/Ta (solid square symbols).

The experimental results presented and discussed in this section are of various electrocatalytic materials used in the PEM electrolyzer. In this investigation, a ribbon mesh anode catalyst structure was placed directly between the GDL and Nafion membrane. Using the RuO_2_ NR-decorated catalyst structure increased the charge transfer coefficient, α. The RuO_2_ NRs provided a means of lowering the potential barrier to the loss of electrons from the quantum well of the hydrogen atom. This change in the square well potential is caused by the enhanced electric field produced by the RuO_2_ NRs. This investigation shows the enhanced electrocatalytic properties caused by the RuO_2_ nanorods. The water electrolysis *J*–*V* plot shown in Figure 6 is followed by a summary of calculated parameters in Table 1. In each of these three cases presented in Figure 6, the electrocatalyst material was detached or not optimally bonded to the electrocatalyst layer. Although not optimal for the cell’s performance, it allowed for a comparison of the different electrocatalyst materials.

Different electrocatalytic materials were the only change across the three cases represented in Figure 6 and Table 1. The Tafel analysis results in two parameters: the exchange current density, *J_o_*, and the electronic transfer coefficient, *α*. These parameters, when used in the Tafel equation (Equation (4)), represent the dependence of the measured current overvoltage. The exchange current density, *J_o_*, was previously defined as the number of available surface sites on the catalyst surface; the electronic transfer coefficient, α, is related to an electron escaping the quantum square well. The influence that an enhanced electric field has on the charge transfer was described above.

The consistency of the size and shape of the ribbon mesh anode catalyst structure somewhat accounts for the similar exchange current density values observed. This analysis focused on the relative charge transfer coefficient for the RuO_2_ NR-decorated catalyst and the Ir/Ta catalyst. At the lower applied voltages, the Ir/Ta electrocatalyst material appeared to have more influence. At higher applied voltages, around 2.2 V, the influence of the RuO_2_ NRs became greater.

The value of the electronic transfer coefficient, *α*, resulting from the RuO_2_ nanorod electrocatalyst material was double that of the Ir/Ta electrocatalyst sheet. An increased *α* is representative of an increase in electron movement across the energy barrier aided by an enhanced electric field effect. This effect became more significant as the applied voltage across the anode and cathode of the PEM electrolyzer increased. The region surrounding the exterior of the RuO_2_ NRs provided an enhanced electric field effect, which ultimately increased the cell performance. The greater electric fields surrounding the RuO_2_ NR-decorated catalyst material increased the charge transfer coefficient, *α*. At the lower applied voltages, the resistance of the nanorod controlled the performance. More specifically, it was the resistance of the interfacial oxide that existed at the interface between the RuO_2_ nanorod and the substrate upon which it was initially grown, i.e., the Telpro MMO ribbon mesh. Growth of the RuO_2_ NRs using this growth technique, i.e., reactive sputtering, can only be performed on a substrate that naturally forms a native insulating oxide nucleation site [14].

**Figure 7:**
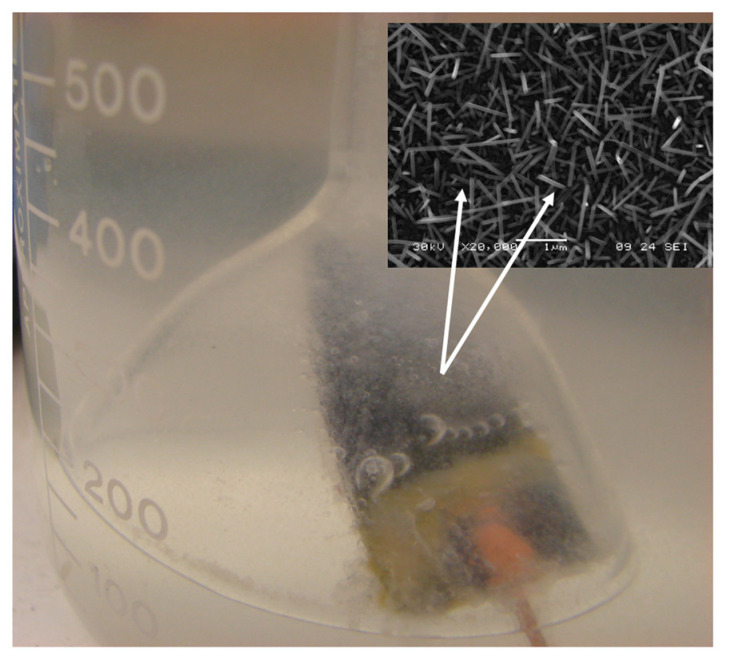
Photo of bubble formation on RuO_2_ nanorod decorated surface (Si wafer). The nanorods are located on the surface of the wafer (see insert).

It is assumed that the nanorod nano-electrode is surrounded by a low-density gaseous water medium, i.e., microbubbles, and not just liquid water, as shown in Figure 7. This is further assured by the use of a properly functioning GDL.

**Figure 8:**
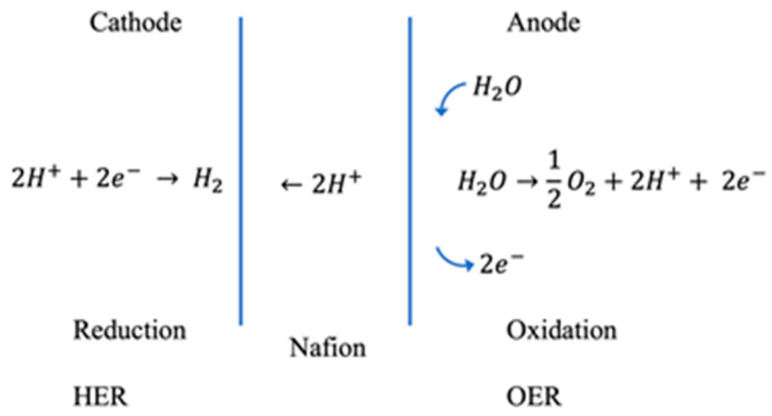
Schematic of PEM electrolyzer assembly and process. The creation of the *H^+^* occurs on the anode side of the PEM electrolyzer.

In a PEM electrolyzer, it is known that the oxidation reaction or the oxygen evolution reaction, OER, occurs on the anode side of the MEA. A schematic of these chemical processes occurring in the PEM electrolyzer is shown in Figure 8. 

It is proposed that the medium surrounding the nanorod consists initially of trapped microbubbles and later generated *O_2_* gas. The Debye length and dielectric constant in liquid water would prevent the creation of a significant electric field; however, in the rarified medium of the bubbles, it is possible. The atomic hydrogen present will be available for the electro-catalyzed ionization to yield *H^+^*.

**Figure 9:**
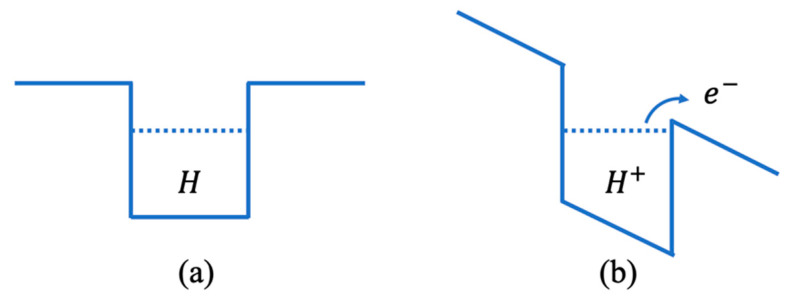
Square well quantum model for *H* atom in OER at the anode: (**a**) in equilibrium; (**b**) in the presence of a high electric field.

A sketch of the proposed one-dimensional square quantum well diagram is shown in Figure 9. The high **E**-field surrounding the nanorod is depicted as a slanted square well in Figure 9b. The slanted square well results in a reduction in the energy barrier to ionization of the *H* atom.

**Figure 10:**
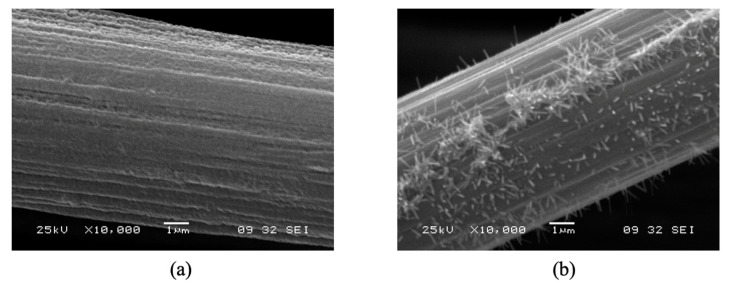
RuO_2_ NRs grown on (**a**) commercially available Toray paper and (**b**) Toray paper with a thin thermally evaporated Ti (TiO_2_) layer.

The RuO_2_ nanorod-decorated ribbon mesh anode catalyst structure has an interfacial oxide layer between the RuO_2_ nanorods and the ribbon mesh anode structure. To fully realize the potential benefits provided by the RuO_2_ nanorods as a catalyst material, the resistance caused by the presence of this interfacial native oxide must be decreased or eliminated. There are five possible directions worthy of future investigation:

1. Post-growth annealing of the RuO_2_ nanorods in a reducing atmosphere;
2. A lower temperature process to grow RuO_2_ nanorods;
3. Replacement of the use of RuO_2_ nanorods altogether with another conducting nanorod with similar catalytic properties to RuO_2_, such as graphite nanorods;
4. Use of a micromachined surface that contains sharp abrupt edges and features;
5. An attempt was made to grow RuO_2_ nanorods on a conducting fabric surface, i.e., Toray paper, with and without a thermally evaporated Ti layer, as shown in the SEM images below (Figure 10). Neither configuration has been tried in the PEM electrolyzer.

## 4. Conclusions

The implementation of RuO_2_ nanorods as an anode electrocatalyst in a PEM water electrolyzer can enhance the overall cell performance as the applied voltages increase. The analysis of recorded data representing the operation of the cell provides deeper insight into the physical processes occurring. The abrupt geometry of the RuO_2_ nanorods contributes to the creation of high electric field regions near the nanorods, similar to the behavior of a lightning rod. This high-field region modifies the quantum barrier surrounding the hydrogen atoms in the electrolyte and lowers the barrier to ionization.

Ideally, what is needed is to produce an electrocatalyst anode material that includes sharp, abrupt nanofeatures. Additional work will be needed to make RuO_2_ nanorods on a more conductive material. The interfacial oxide that forms on the growth surface adds resistance. A new low-temperature process to grow RuO_2_ nanorods on the Nafion membrane or a material that is in direct contact with the Nafion layer is needed. Another option is to pursue other nanorod materials such as graphite nanorods of other conducting nanorods. Another possibility is to provide a micromachined layer on the anode electrocatalyst surface that has abrupt features that can support the creation of high-electric-field regions in the vicinity of the microstructure.

The enhanced electrocatalytic properties of materials decorated with RuO_2_ nanorods were observed experimentally. The overall result of the investigation described above is a new route to potentially enhance the activity of electrocatalyst materials used in a PEM electrolyzer cell.

## Figures and Tables

**Table 1:** Overpotential parameter values.

Overpotential Parameter	Exchange Current Density *J_o_* (mA/cm^2^)	Charge Transfer Coefficient *α* (eV^−1^)	Limiting Current Density *J_l_* (mA/cm^2^)
Cell Configuration			
Bare anode MEA with RuO_2_ nanorod catalyst ribbon	1.7240	0.1067	94.1262
Bare anode MEA with Ir/Ta catalyst sheet	1.7240	0.0587	94.1262
Bare anode MEA with Ti catalyst ribbon	1.7240	0.0335	94.1262
